# CLSM-Guided Imaging for Quantifying Endodontic Disinfection

**DOI:** 10.3390/antibiotics13010054

**Published:** 2024-01-04

**Authors:** Rebecca Mattern, Sabrina Ernst, Sarah Böcher, Andreas Braun, Johannes-Simon Wenzler, Georg Conrads

**Affiliations:** 1 Department of Operative Dentistry, Periodontology, and Preventive Dentistry, Rheinisch-Westfälische Technische Hochschule (RWTH) University Hospital, Pauwelsstrasse 30, 52074 Aachen, Germany; rebeccamattern@outlook.com (R.M.); sboecher@ukaachen.de (S.B.); anbraun@ukaachen.de (A.B.); jwenzler@ukaachen.de (J.-S.W.); 2 Division of Oral Microbiology and Immunology, Department of Operative Dentistry, Periodontology, and Preventive Dentistry, Rheinisch-Westfälische Technische Hochschule (RWTH) University Hospital, Pauwelsstrasse 30, 52074 Aachen, Germany; 3 Confocal Microscopy Facility, Interdisciplinary Center for Clinical Research IZKF, Rheinisch-Westfälische Technische Hochschule (RWTH) University Hospital, Pauwelsstrasse 30, 52074 Aachen, Germany; sabernst@ukaachen.de

**Keywords:** endodontic treatment, root canal disinfection, *Enterococcus faecalis*, CLSM, LIVE/DEAD-staining, bacterial penetration depth, irrigation depth, sodium hypochlorite, EDTA, EDDY

## Abstract

Elimination of microbes in the root canal system is crucial for achieving long-term success in endodontic treatment. Further efforts in study design and standardization are needed in order to improve the validity and comparability of in vitro results on endodontic disinfection procedures, in turn improving clinical outcomes. This study optimizes two models at all steps: tooth selection, pretreatment, inoculation method (by growth or centrifugation), and confocal laser scanning microscopy (CLSM)-guided imaging of LIVE/DEAD-stained specimens. Individual anatomical conditions lead to substantial differences in penetration depth. Sclerosis grading (SCG), a classification system introduced in this study, provides information about the sclerosis status of the dentine and is helpful for careful, specific, and comparable tooth selection in in vitro studies. Sonically activated EDTA for the pretreatment of roots, inoculation of *Enterococcus faecalis* in an overflow model, 3–4 weeks of incubation, as well as polishing of dentine slices before staining, led to advances in the visualization of bacterial penetration and irrigation depths. In contrast, NaOCl pretreatment negatively affected performance reproducibility and should be avoided in any pretreatment. Nonsclerotized teeth (SCG0) can be used for microbial semilunar-shaped inoculation by centrifugation as a “quick-and-dirty” model for initial orientation. In conclusion, CLSM-guided imaging for quantifying endodontic infection/disinfection is a very powerful method after the fine-tuning of materials and methods.

## 1. Introduction

Eliminating bacteria in the root canal system is crucial for achieving long-term success in endodontic treatment, as the persistence of bacteria is the main reason for failure [1,2]. As many as 10^2^–10^7^ bacterial cells may be present in infected root canals, their accessory side canals, and anastomoses. They penetrate deep into the infected tubules of the root dentine [3]. However, on average, only 80% of the bacteria can be eliminated by chemical-mechanical treatment [4,5]. Sodium hypochlorite (NaOCl) is the most commonly used disinfectant in endodontics due to its antibacterial and tissue-dissolving properties [6,7]. Various studies have shown that difficult-to-treat Gram-positive, recurrence-associated bacteria such as *Enterococcus faecalis* (*E. faecalis*) can colonize the dentinal tubules to a depth of 1350 µm, whereas NaOCl penetrates only to a depth of 300 µm [4,8,9,10]. *E. faecalis*—among other bacteria—has consequently been shown to be associated with many cases of failed endodontic treatment [11,12,13,14]. Irrigation methods have therefore been the focus of numerous in vivo and in vitro studies in endodontics for many years. A major challenge for the reproducibility and comparability of such studies is the wide variation between individual teeth in terms of anatomy, size, and the shape of the canal [15]. In particular, the patient’s age and the resulting degree of dentinal sclerosis are not usually factored into the evaluation of results after oversimplified experimental setups. In addition, placing a predictable (saturated) and relevant number of bacteria into the tubule system in vitro continues to be a challenge [16]. To validate rinsing methods, however, artificial bacterial inoculation must be of high density, uniformity, and depth [17,18]. Further efforts in study design and standardization are needed in order to improve the validity and comparability of in vitro results with endodontic disinfectants and disinfection procedures, in turn improving clinical outcomes.

Most studies use an invasion model based on culture methods, in which model bacteria invade and multiply in dentinal tubules through natural biofilm formation in various media within a certain period of time in defined atmospheric conditions. To maximize the depth of bacterial invasion, the root samples in most studies are pretreated with NaOCl and ethylenediamine tetraacetic acid (EDTA) prior to incubation in order to ensure tubule access [9,19,20]. However, as we and other authors have noted, this pretreatment process fundamentally alters the dentine structure, making the comparison of subsequently performed procedures difficult. Ma et al. instead used a “quick-and-dirty” model in which the bacteria were just flung into the tubules by centrifugal force [15].

A number of studies have already visualized bacteria in dentinal tubules using confocal laser scanning microscopy (CLSM), which offers the advantage of allowing LIVE/DEAD and other functional staining in addition to three-dimensional imaging. LIVE/DEAD staining allows visual identification and differentiation of live and dead bacteria in infected dentine, as the dye SYTO 9 penetrates all bacteria (intercalating into every DNA/dsRNA fragment), while propidium iodide only reaches the DNA/RNA of membrane-defective or dead cells [21,22,23].

The aim of this feasibility study was to optimize a CLSM-based method for evaluating the quality of different irrigation regimes in the disinfection of root canals. For this purpose, different parameters (inoculum, medium, incubation time) of two inoculation models (overflow culture and centrifugation) were tested to quantitatively evaluate the number, penetration depth, and viability of *E. faecalis* as an indicator organism. Both the infection and disinfection of the dentinal tubules can only be reproducibly assessed after the dentine sclerosis grade (SCG) has been determined. This can be carried out using an SCG scale (0–3), which we also present in this study. In addition, the extent to which pretreatment with NaOCl, a standard in test sample preparation, affects the results was investigated. After fine-tuning various parameters, CLSM is very useful for obtaining radiation-free (in contrast to SEM), precise, and informative insights into the microanatomy of the dentine structure, the biofilm invading it, and the positive and negative effects of any treatment protocol tested.

## 2. Results

### 2.1. Tooth Preparation and Pretreatment

**Figure 1:**
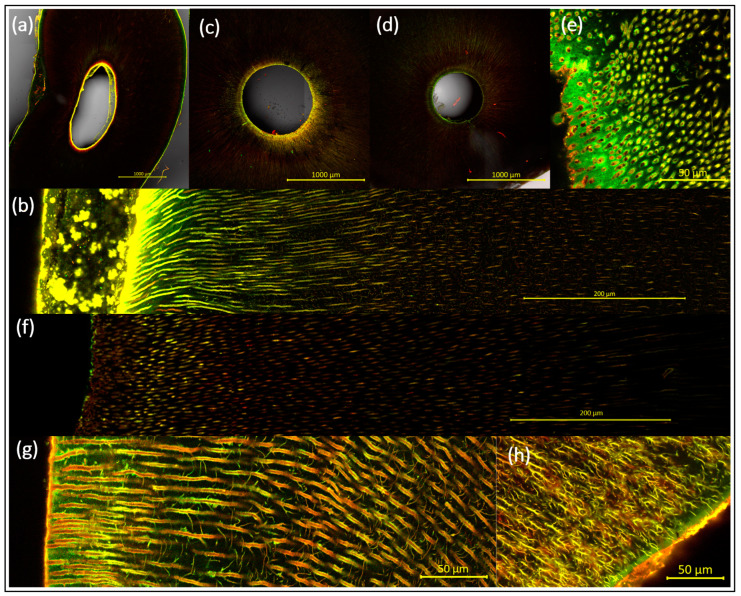
Uninoculated root canals with sections at various magnifications and after LIVE/DEAD staining. The edge of the pulp cavity and the outer tooth surface can be seen as fluorescent lines; before (**a**,**b**) and after (**c**–**h**) pretreatment with either Pr1 ((**c**), with NaOCl) or Pr2 ((**d**–**h**), without NaOCl). The smear layer, patient-borne microorganisms, and odontoblasts are stained due to intracellular/extracellular DNA/RNA and are thus visualized inside the root canal and tubules even before inoculation ((**a**), overview with a 3 × 3 tile scan; (**b**), detailed, with a 9 × 2 tile scan). Before microbial inoculation, it had to be ensured that the dentinal tubules were open, clear, and unimpeded/debris-free. Details are shown of pretreated tubules sectioned transversely (**e**) or longitudinally (**f**,**g**). Many details of the root histology become visible here with this preparation and staining protocol—for instance, the mantle dentine with branched tubules at the root surface (**g**,**h**). In the latter images, for better visualization, the contrast was increased in comparison with all the other images.

Figure 1 depicts uninoculated root canals after LIVE/DEAD staining at various magnifications to provide an insight into the initial situation without any added bacteria. Root canals before any instrumentation can be seen in Figure 1a,b. A smear layer, microorganisms from the donor patient, odontoblasts, and their microvilli that extend through the dentinal tubules can be seen on the root canal walls of these untreated samples. All cells and cell debris are stained and thus visualized due to intracellular/extracellular DNA/RNA. After pretreatment with Pr1 (Figure 1c, with NaOCl) and autoclaving, the cells and debris are removed, but some fluorescence is still detectable. In contrast, after pretreatment with Pr2 (Figure 1d–h, without NaOCl) and autoclaving, the orifices of the dentinal tubules were completely opened, clear, unimpeded, and debris-free as a precondition for even inoculation. Many details of the root histology become visible with this preparation and staining protocol—for instance, the mantle dentine with branched tubules at the root surface (Figure 1g,h).

### 2.2. Assessment of Sclerosis Grading (SCG)

**Figure 2:**
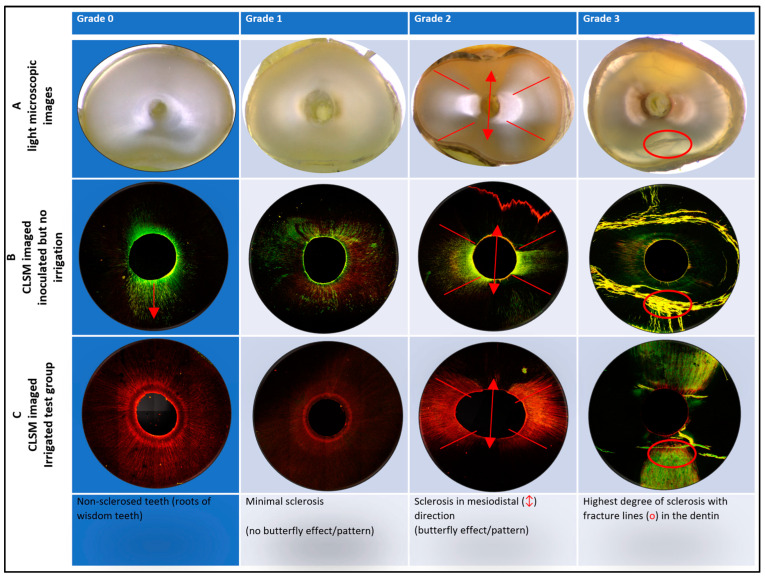
Sclerosis grading (SCG) from 0 to 3 with examples. Transverse slices of roots with increasing degrees of sclerosis presented by light stereomicroscope images (**row A**) and CLSM-guided imaging, the latter either without irrigation (**row B**) or with sonically assisted (EDDY) irrigation (**row C**). For best results, the pretreatment protocol Pr2 (without NaOCl) was applied. Inoculation was carried out with 15 µL of 10^6^ CFU *Enterococcus faecalis* in 200 µL 0.5 × Müller–Hinton broth, followed by 3–4 weeks of incubation in the case of SCG1–3 (**process A**) or centrifugation in the case of SCG0 (**process B**). The arrow ↓ indicates the centrifugal force. **Grade 0**: no sclerosis; radial inoculation pronounced in the direction of centrifugal force. **Grade 1**: little or no butterfly pattern. **Grade 2**: the butterfly-like pattern of oral-vestibular bacterial invasion (visualized by the red lines) and—in contrast—the dark area of sclerosis in a mesiodistal direction can be seen ↕. **Grade 3**: the multiple fracture lines should be noted (red circles). Vital cells are stained green by SYTO 9, whereas membrane-defective and/or dead bacteria—as well as extracellular DNA/dsRNA—are stained red by propidium iodine. Fracture lines show yellow-green fluorescence, probably due to dye precipitation and/or reflections.

Since sclerosis of the dentine tubules was observed to be an important factor for reproducible inoculation/disinfection, classification and sorting of appropriate/inappropriate material was carried out, which is introduced as follows (Figure 2). Wisdom teeth were designated as SCG0, as they show the lowest degree of sclerosis. Minimal sclerosis without a butterfly pattern was labeled as SCG1. As soon as a clear butterfly pattern became visible (with sclerosis in the mesiodistal direction), these specimens were defined as SCG2. The ultimate degree of sclerosis, resulting in fracture lines, was defined as SCG3. Each slide for the test specimens was allocated to a sclerosis grade before CLSM imaging.

### 2.3. Results of Specimen Inoculation and Quantification of Bacterial Invasion Depth

**Figure 3:**
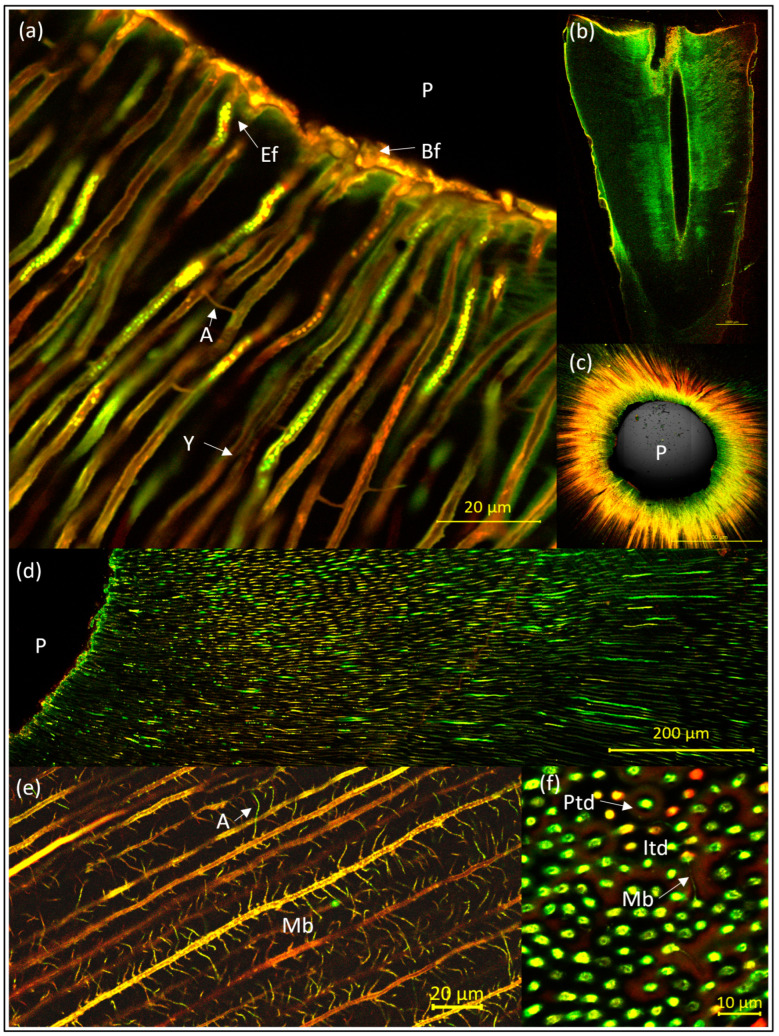
Inoculated root canals with sections at various magnifications and after LIVE/DEAD staining. Inoculation was carried out after pretreatment of the roots with either Pr2 ((**a**–**c**), without NaOCl) or Pr1 ((**d**–**f**), with NaOCl). The model organism applied was *Enterococcus faecalis,* incubated for 3–4 weeks after inoculation. In (**a**) the following details can be observed: root canal wall of pulp (P) with attached fresh biofilm (Bf) (orange layer), intratubular cells of *E. faecalis* (Ef), anastomoses (A), and Y-ramification (Y). (**b**) An example root in longitudinal section (scale bar 1000 µm). (**c**) Cross-section showing staining of the entire dentin disc after pretreatment with NaOCl (A-Pr1) (scale bar 1000 µm). (**d**) Dentine tubules sectioned longitudinally with patterns of vital *E. faecalis*. (**e**) Details of the mantle dentine with microbranches (Mb) that have anastomosed (A) with other microbranches. For better visualization, the contrast was increased in comparison with all other images here. (**f**) Detailed view of dentine tubules with *E. faecalis* in dentine sectioned transversally. Peritubular dentine is stained reddish (Ptd) in contrast to the unstained intertubular dentine (Itd).

**Figure 4:**
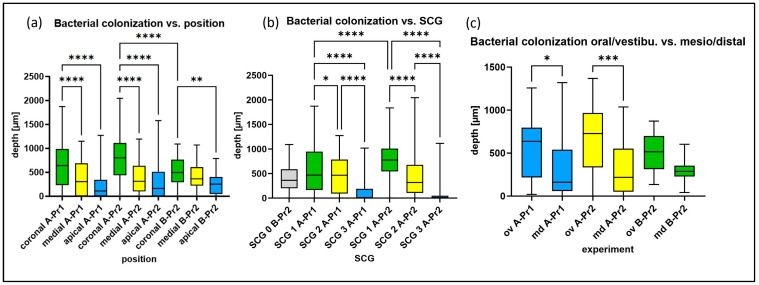
Statistical analysis of bacterial colonization depths (in µm) depending on three parameters, with means and 25–75% quantiles: (**a**) Dependency on coronal-apical position. (**b**) Dependency on sclerosis grade. As all SKG0 teeth were solely used for the centrifugation model, statistical comparison with other teeth is not permitted. (**c**) Dependency on an oral-vestibular (ov) versus mesiodistal (md) orientation. All results are split by pretreatment protocol (Pr1/2) and experimental model (A/B). * < 0.05, ** < 0.01, *** < 0.001, **** < 0.0001.

An incubation period of 3–4 weeks proved to be optimal, as 1–2 weeks lead to a low and suboptimal penetration depth, while longer incubation did not improve the results. It should be emphasized that the difference between 3 and 4 weeks of incubation was minor and depended on the sequence of medium refreshing, which again depended on the sequence of working days/weekends. Clear and evenly distributed fluorescence was detected in nonsclerotized areas of all specimens after incubation and preparation (Figure 3). At higher magnification, details of biofilm-like structures were observed on the root canal walls and *E. faecalis* cells lining up inside the dentine tubules (abbreviated Ef in Figure 3a). The maximum measured colonization depth was about 2300 µm. The consistently highest mean penetration depth was achieved in coronal samples of SCG1, pretreated with Pr2 (EDTA-EDDY only) and grown for 3–4 weeks in the overflow model (A-Pr2). The bacterial penetration depth significantly decreased from coronal to apical (in the case of A-Pr1 sections, it differed by *p* < 0.0001; in A-Pr2 by *p* < 0.0001, in B-Pr2 by *p* < 0.01) (Figure 4a). In coronal samples, A-Pr2 had significantly greater colonization depths than B-Pr2 (*p* < 0.0001). From SCG1 to SCG3, the penetration depth of the bacteria also decreased significantly (A-Pr1: SCG1 > SCG2 with *p* < 0.05, SCG2 > SCG3 with *p* < 0.0001, SCG1 > SCG3 with *p* < 0.0001; A-Pr2: SCG1 > SCG2 with *p* < 0.0001, SCG2 > SCG3 with *p* < 0.0001, SCG1 > SCG3 with *p* < 0.0001) (Figure 4b).

**Figure 5:**
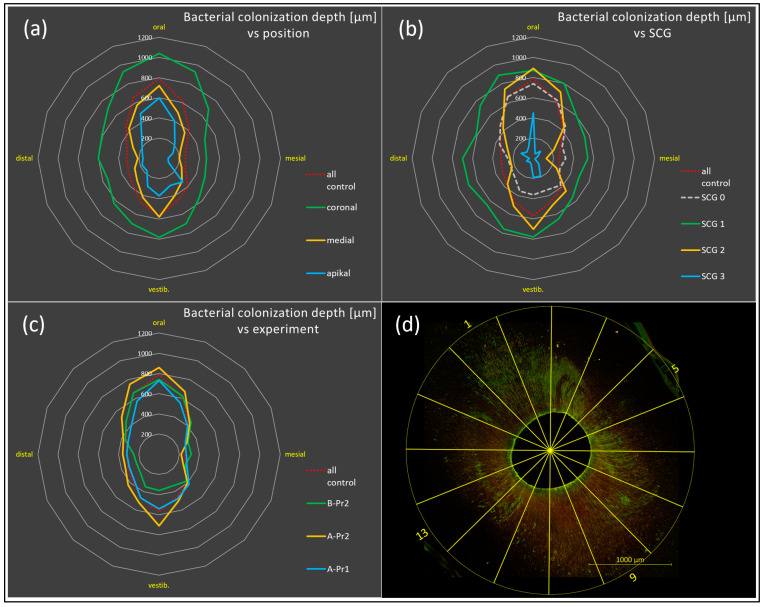
Radar charts showing dependency of bacterial colonization depth (in µm) on position: apical to coronal (**a**), sclerosis grade (SCG) (**b**), and three experimental settings, combining process A/B with pretreatments Pr1/2 (**c**). Overall, A-Pr2 produced the greatest penetration depths in less sclerotized teeth in the coronal area. For all experiments, penetration in the oral-vestibular (ov) direction was significantly greater in comparison with the mesiodistal (md) areas, except for the centrifugation experiment as applied to nonsclerotized teeth only. (**d**) Example CLSM image showing the 16 segments (arcs) of a circle of measurement.

In contrast, Pr2-treated samples (with NaOCl) together with inoculation by centrifugation (process B) showed the lowest mean penetration depths (Table 1). SCG1 samples showed significantly greater penetration depths with A-Pr2 than with A-Pr1 (*p* < 0.0001) (Figure 4b). During the 3–4 weeks of natural growth of bacteria into the tubules (overflow model and process A), the stained areas (penetration depths of the last detectable bacterial cells) in the oral-vestibular (ov) direction became significantly larger in comparison with the mesiodistal (md) areas (for A-Pr1 with *p* < 0.05, for A-Pr2 with *p* < 0.001 (Figure 5 with statistical analysis in Figure 4c)). This deviation was most evident in SCG2 and SCG3 teeth (Figure 5b; see waisted lines in orange and blue). When the centrifugation process (B) was applied to nonsclerotized teeth, there was no oral-vestibular enlargement. However, the penetration depth here was increased in the direction of the last centrifugation force instead.

### 2.4. Quantification of Treatment Efficacy

**Figure 6:**
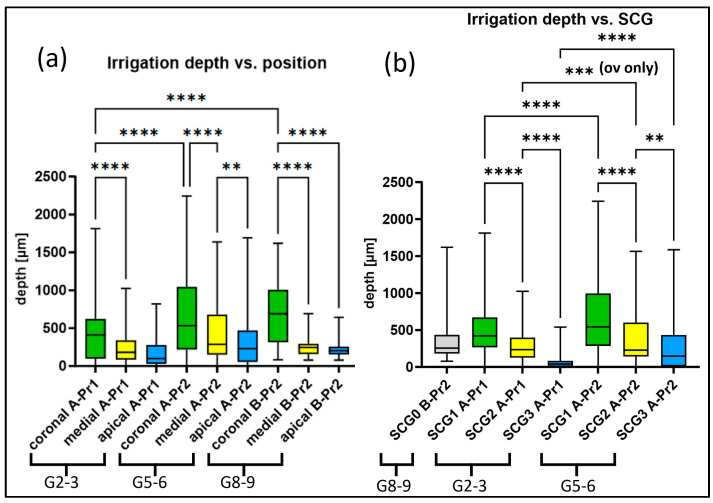
Statistical analysis of irrigation depths (in µm) depending on two parameters: (**a**) position from apical to coronal, (**b**) sclerosis grade (SCG); means and 25–75% quantiles are given for both (compare with Figure 4). ** < 0.01, *** < 0.001, **** < 0.0001. ov—oral-vestibular.

The ultimate goal of this study was to optimize the imaging of endodontic treatment effects (conventional rinsing versus sonic [EDDY]-activated rinsing, both versus controls). Very similar to the colonization depth, the irrigation depth also decreased highly significantly from coronal to apical in all experimental groups (Figure 6) (G2–3, G5–6, G8–9; with G1, 4, 7 as corresponding controls). However, G2–3 (A-Pr1) had significantly lower irrigation depths in coronal slices than G5–6 (A-Pr2) and G8–9 (B-Pr2) (Figure 6a). The irrigation depth decreased from SCG1 to 3 (G2–3 (A-Pr1): *p* < 0.0001; G5–6 (A-Pr2): SCG1 > SCG2 *p* < 0.0001, SCG2 > SCG3 *p* < 0.01), with the exclusion of SCG0, as it was only used for centrifugation. It turned out that the irrigation depth was significantly greater in all sclerosis grades in Group 5–6 (A-Pr2) than in Group 2–3 (A-Pr1) (SCG1, SCG3: *p* < 0.0001, SCG2: *p* < 0.001) (Figure 6b).

**Figure 7:**
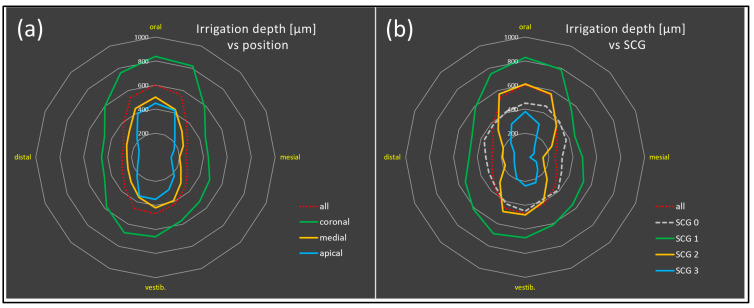
Radar charts showing dependency of irrigation depth (in µm) on position ((**a**), apical to coronal) and sclerosis grade (SCG) ((**b**), SCG0–3), which both have a highly significant impact (compare with Figure 6).

Both bacterial colonization and irrigation depth were dependent on the same parameters (compare Figure 5 and Figure 7). It turned out that when the bacterial colonization depth increased, the irrigation depth also increased.

## 3. Discussion

Optimization of the processes of tooth selection (e.g., age of donor, determination/assessment of SCG after cutting the crown, Figure 2), pretreatment (Pr1, Pr2), choice of the inoculation process (A, overflow model, versus B, centrifugation model), and subsequent procedures (cutting, polishing, staining, microscopy) improved the image quality substantially as a precondition for the unambiguous visualization and quantification of bacterial penetration depth.

The individual steps will be discussed in detail.

The depth of microbial colonization/penetration must be reliably and reproducibly deeper than the subsequent treatments to be tested. In vivo, bacterial cells penetrate slowly but constantly over years and can reach depths of almost 2500 µm. It is important to mimic this process in a reproducible manner within a short (experimental) period of 4 weeks, for example. The latter is difficult, as no individual tooth is like any other. In order to create sufficiently large openings on the tubules, endodontic files with a taper of 0.05 were used to cut the dentinal tubules as obliquely as possible.

All models for evaluating/optimizing a treatment need positive and negative controls. Ensuring the adequacy of the experimental model, no bacterial fluorescence was observed in the negative control group (uninoculated) after pretreatment. In most studies, samples have been pretreated with EDTA and NaOCl, both with or without sonic activation, to remove the smear layer and to open and enlarge the tubules before incubation [9,19,20]. However, freshly inoculated positive controls, pretreated by Pr1 (with NaOCl), showed a halo of red staining, despite the fact that bacterial cells were still alive. This red fluorescence of positive controls was not present in pretreated Pr2 samples (without NaOCl). The red stain, which was radially distributed (halo-like) like a diffusion zone or chromatogram, could be explained by the activity of NaOCl lysing each cell and releasing DNA/RNA, which subsequently evidently diffused into any accessible area and especially into (soft) zones with reduced mineralization. In this context, NaOCl seems to cause ultrastructural alterations and probably collagen denaturation and destruction, weakening the peritubular area. It has previously been demonstrated that NaOCl negatively affects the physical and mechanical properties of dentine—e.g., by decreasing flexural strength and microhardness or by causing ultrastructural alterations in the elastic modulus and inorganic content, as well as in the organic/inorganic ratio of dentine [24,25,26,27]. A widening of the peritubular area could also lead to an exaggerated penetration depth of the bacteria in the model. In conclusion, it appears here so far that NaOCl—a gold standard for chemomechanical root canal preparation—has a substantial influence on the experimental model and should be avoided. In other words, to demonstrate the effect of NaOCl, or any other endodontic therapy, pretreatment with NaOCl is counterproductive. In addition, our results generally showed a greater bacterial penetration depth after Pr2 (without NaOCl), although not always significantly, due to the influence of cofactors such as position (apical-coronal) and SCG, among others (Figure 5a,b). These cofactors can easily lead to underpowered statistical analysis, which is a limitation of our study here, where many protocols had to be tested. In any case, pretreatment only with EDTA and activated by EDDY appears to be superior for evaluating endodontic investigations.

The Gram-positive, facultative anaerobic bacterium *E. faecalis* was chosen as the test organism here because of its strong association with failed endodontic treatments [11,12,13,14]. *E. faecalis* is well adapted to the prevailing environment inside the dentinal tubules. It is able to invade the dentinal tubules and adhere to type I collagen fibers [28]. Various studies have already shown an invasion depth of *E. faecalis* of 1000–1350 µm [4,8,9,10]. In the present study, vital (green) cells were demonstrated to penetrate even deeper than 2300 µm. Other reasons for choosing *E. faecalis* were its wide use in endodontic disinfection studies and its physical and environmental resistance (robustness) during handling, centrifugation, and long-term incubation. In addition, the coccoid shape of the enterococci may facilitate entry into the dentine tubules during centrifugation [15]. However, reproducible quantification of bacterial cell numbers and viability before and after disinfection remains a challenge in endodontic research. In the first instance, placing an appropriately large predictable/reproducible number of bacteria deep into the tubule system in vitro remains difficult [9,16]. In contrast to the commonly used cultural methods for incubation, Ma et al. used centrifugal forces to deeply spin bacteria into the maximum number of tubules in a dentine block [9,15]. We modified and optimized both methods of incubation, using growth in an overflow model and centrifugation. The overflow model developed had advantages in handling, as sealed outer root surfaces guarantee that bacteria can only enter the dentine tubules via the inner root canal surface and not from external fissures or gaps. In addition, this model ensured a reservoir and sufficient flow of nutrients toward the bacteria and also of end products, including toxins, from the bacteria in order to prolong or maintain survival for up to 4 weeks. It should be noted here that *E. faecalis* grew more sustainably/reproducibly and thus survived longer in a nutrient-poor medium such as 0.5 × MH than in the commonly used (very nutrient-rich) BHI, which has high and rapid substrate consumption and end-product production. Deep bacterial penetration was only rarely seen after 1–2 weeks. Over all tubules, only a shallower penetration depth was achieved. In contrast, an incubation period of 3–4 weeks resulted in a homogeneous and dense presence of bacteria in deep areas of dentine in most (and even different) specimens, creating a mature biofilm and mimicking a naturally infected tooth, whereby a single model organism of any kind can never simulate the complexity of multi-species colonization as it exists in vivo. In comparison with the overflow model, the advantage of the centrifugation model was the short processing time (a few hours), followed by a short incubation time of only 24 h. By repeating the centrifugation process three times in different positions (−45°, 0°, +45°), an attempt was made to achieve the largest possible colonization angle. However, as the last centrifugation affects all others before, the centrifugal force cannot lead to a radial penetration pattern in all directions, as in the overflow model. Consequently, only a small tubule area (in a semilunar shape) could be used for experiments here. In addition, precise and reproducible fixation of specimens in the oral-vestibular or mesiodistal direction is difficult. It is also uncertain whether or not centrifugation forces, as an additional stress factor for the cells, affect their resistance to disinfectants, even if they have a period of 24 h to recover after centrifugation [15]. Most importantly, the centrifugation method only works with SCG0 teeth, which severely limits the selection of teeth. As demonstrated by our results, the penetration depth of samples grown for 3–4 weeks was significantly greater than in those inoculated by centrifugation force and with a short 24 h incubation period, although the latter had a lower SCG. However, to be able to significantly compare the penetration depths of the two protocols (methods), the number of teeth inoculated using the centrifugation method will need to be increased in further investigations. In summary here, the culture model is preferable to the centrifugation model for the many reasons mentioned above.

To the best of our knowledge and through the comparison of many published images, our overflow method, especially in the A-Pr2 variant, led to CLSM images of the microanatomy of dentine and resident bacteria that have rarely been seen before in such detail. The image resolution was mainly improved by polishing the samples, which prevents unevenness on the surface leading to artificial fluorescence (due to reflections and surface/precipitation phenomena), which may be confused with true DNA/RNA-based fluorescence. It was confirmed that the bacteria were still located in the dentinal tubules after polishing. As seen with control specimens, the intensity of the background fluorescence was minimal in the tubules and did not interfere with the fluorescent signal generated by the bacteria. However, despite polishing, unexplained artificial fluorescence sources remained—e.g., due to fracture lines in the dentin. Nevertheless, the method enabled the examination of microstructures of the dentine in detail. For instance, with the 40 × magnification lens, anatomical features such as longitudinally and transversely sectioned dentinal tubules, Y-ramifications, microbranches, anastomoses, and odontoblasts could be visualized. It was possible to show anastomoses even between microbranches, for example. This observation is consistent with the SEM images presented in a previous study [29]. The branching patterns showed an intricate and rich canalicular anastomotic system crisscrossing the intertubular dentine and branching more and more peripherally. The number of branches in the dentinal tubules was particularly high in those areas in which the density of the tubules was low [29]. It is noteworthy that SCG0 teeth tend to have more branching than SCG1–3, thus representing sclerotized teeth. Further investigations will be needed in order to confirm this finding. In addition to the transverse slices, only as examples, longitudinal slices (cuts) were obtained to illustrate the effect of our methods more three-dimensionally. However, due to the butterfly pattern, it is difficult to cut the teeth all in the same plane in longitudinal sections. These longitudinal overview images are therefore not ideal for comparative studies, as areas with the most tubules (e.g., in nonsclerotized areas) can easily be missed. Detailed knowledge of dentine structure, especially the histology of dentine and distribution of lateral canals and tubules, is essential for understanding dentine permeability and interpreting data from endodontic treatment studies. This is clinically important, as the penetration (paths) of bacteria and their antigens/toxins may not be equivalent over all areas of a particular tooth to be treated [29].

Various microscopic techniques have been used to assess the bacterial colonization of dentine, including stereomicroscopy [30], SEM [29], transmission electron microscopy (TEM) [31], and CLSM [9]. The fact that the fluorescent substances visualized by CLSM can penetrate as deep as 10 µm below the surface of a particular sample is a huge advantage in comparison with TEM/SEM or simple light stereomicroscopy [19]. Three-dimensional imaging in z-stacks provides a complete three-dimensional view of the course of tubules, which is particularly important as they do not run in a straight line. Another drawback of SEM imaging is that it does not help to determine the viability of the bacteria, whereas CLSM, along with LIVE/DEAD staining, provides information about the vitality of the bacteria and the impact of disinfecting substances. Furthermore, and as shown here for the first time, CLSM may provide information about the exact dentine density, due to RNA/DNA diffusion into demineralized (collagen-free) spaces, revealing destruction.

It became apparent, however, that the penetration depths in our samples varied widely, even though the experimental conditions were the same. It turned out that inalterable anatomical factors appear to have a strong effect on the colonization depth into the tooth. The reason for this may be related to dentine sclerosis, which increases with age along the tubules located on the mesial and distal sides of the canal lumen [32,33]. This phenomenon is known as the butterfly effect. It leads to a unique pattern of sclerotic dentine that produces a characteristic butterfly shape in transverse sections of the roots. The dentinal tubules in the mesiodistal direction have a lower density than those in the vestibular-oral direction, which corresponds to the butterfly’s wings [34]. In the present study, this effect is reflected in the colonization pattern of bacteria and the penetration depth of the rinsing solution. Bacterial colonization was significantly deeper in the vestibular-oral direction in comparison with the mesiodistal direction. This finding is consistent with a previous study [19]. For samples that show the butterfly effect, the measurement position is essential. A simple/single mean value, or the median over all the positions, is definitely not a valid way of evaluating success, as it shows a wide standard deviation. Instead, a total of 16 measurement positions (sections of a circle, arcs) should be considered individually, as this results in position-dependent mean values that provide omnidirectional information. This is also the reason why transverse slices are preferable to longitudinal slices, as it is very difficult to always cut them exactly along the same vestibular-oral plane. A statistic based on 16 single measurements per specimen was sufficient to demonstrate significant differences. However, statistics based on the area of bacterial colonization (with *E. faecalis* and other test organisms) or the area of anti-infective irrigation as measures might enhance or even advance the findings.

The main limitations—among others—of our study are the low number and unequal distribution of specimens and the usage of a single bacteria indicator species.

To create comparable conditions for further experiments, it is important to quantify and classify anatomical differences. We have therefore developed a sclerosis grading (SCG) system as an innovation here. Previous studies have shown (i) that root sections with the butterfly effect were harder mesiodistally and (ii) that there is a correlation between the butterfly effect and a higher prevalence of vertical root fractures in the vestibulo-oral direction [35,36], with the latter confirmed in the present study. We defined this ultimate level of sclerosis, leading to fracture lines, as SCG3. In all the specimens examined, the SCG increases or at least continues from coronal to apical, as has been observed previously [37].

In summary, individual anatomical conditions are a significant factor that cannot be influenced but can be controlled by specific tooth selection in in vitro studies. Caries-free, unfilled teeth, if possible single-rooted, permanent, and freshly extracted, are standard requirements for describing test specimens. However, it is difficult to draw conclusions regarding the sclerosis status of the dentine. If possible, only teeth of SCG0-2 should be subjected.

## 4. Materials and Methods

The materials, pretreatment procedures, and inoculation procedures are described in detail below.

### 4.1. Tooth Selection and Classification

A total of 150 freshly extracted, caries-free, intact, permanent human teeth with straight root canals were selected. Teeth with previous coronal restorations, root canal treatment, resorption, or incomplete root growth were excluded. While process A used single-rooted teeth, process B used roots of wisdom teeth. The study was conducted in full accordance with established ethical principles (World Medical Association Declaration of Helsinki, version VI, 2002). First, the selected teeth were cleaned by scaler, sterilized by autoclaving, and stored in sodium chloride/sodium azide (NaCl 0.9%, 0.001% NaN_3_, at 7 °C), then decoronated to a 12 mm standard root segment using a rotary diamond saw (Primus cut-grinder, Walter Messner GmbH, Oststeinbek, Germany) with water cooling.

### 4.2. Preparation and Pretreatment Protocols

Tooth preparation and rinsing were carried out using a modified protocol described by Galler (2019) [20]. The root segments were distributed across two pretreatment groups with different protocols (Pr1 and Pr2).

To determine the working length, patency was initially checked with a size 08, 0.02 taper K-file (K-files, VDW, Munich, Germany) until visible at the foramen apicale. Since the distance between the foramen apicale and the foramen physiologicum is 0.89 mm on average, the actual working length was defined by subtracting 1 mm from this length. Thus, a glide path was prepared with hand files from size 08, 0.02 taper to size 25, 0.02 taper. As the root length was defined as 12 mm, the working length thus equaled approximately 11 mm. The root canals were enlarged in a crown-down manner with rotary files (Protaper Gold, Dentsply Sirona, Bensheim, Germany) up to size 50, 0.05 taper. For irrigation during instrumentation, Pr1 used 5% NaOCl (initially at 40 °C, then at room temperature (RT) 20 °C) with a volume of 1 mL between files. In contrast, for Pr2, NaCl was used as an alternative to NaOCl in order to avoid possible prior damage to the dentine before inoculation. Both pretreatment protocols, shown in Table 2, use 27-G side-vented needles (Covidien, Dublin, Ireland) for irrigation.

After pretreatment, the root segments were stored in bidistilled water overnight. Five samples of pretreatment Pr1 and Pr2 were randomly selected as negative controls and examined by CLSM to ensure that the smear layer and unintended (prior) microorganisms were (almost) fully eliminated. All others were subjected to inoculation with *E. faecalis* using two different models and processes, explained below in Table 2.

### 4.3. Overflow Model (Process A)

**Figure 8:**
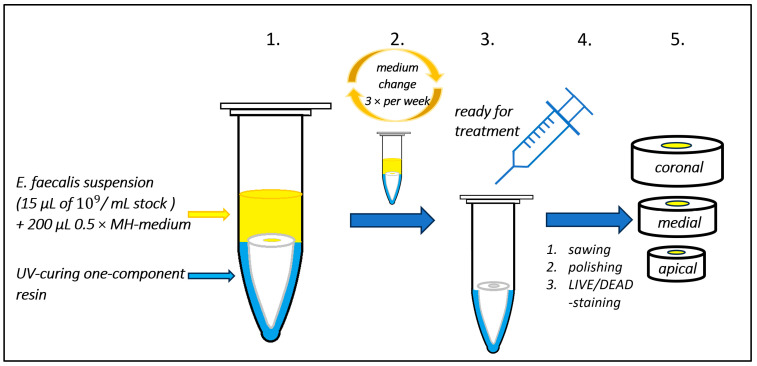
Schematic representation of the overflow model for inoculation of roots and subsequent procedures for treatment evaluation and visualization (process A with steps 1–5). After 3–4 weeks of incubation, the model is ready for testing of endodontic treatment protocols, always against a negative control. For CLSM, roots are cut in three sections (apical, medial, coronal) and LIVE/DEAD-stained. MH—Müller-Hinton broth; UV—ultra-violet.

As an innovation, a new overflow model for inoculating teeth (tubules) inside-out with *E. faecalis* is named and introduced here. The roots were placed in sterile 1.5 mL Eppendorf tubes. The gap between the tube and the root was sealed using UV-curing, one-component resin (Eukitt 4400 LB; Walter Messner GmbH, Oststeinbek, Germany) and cured for 20 min using a light polymerization box (KARLO; Walter Messner GmbH, Oststeinbek, Germany). The resin was used to prevent bacteria from entering via side channels or gaps in the cement outside-in. The upper coronal surface with the root canal entrance remained uncovered. After this manipulation and before inoculation the next day, the fixed root models were sterilized by autoclaving. Root canals (and tubules as far as possible) had to be dried of any water with sterile paper points before inoculation to ensure the access of nutrient-rich medium. The overflow experimental model is shown in Figure 8 and all procedures (steps 1–5, summarized as process A) are described below (Table 2).

Gram-positive *E. faecalis* (strain ATCC 29212), thawed from a frozen stock, was used as the test organism. After initial growth on brain–heart infusion (BHI) agar plates, one or two colonies were used to inoculate 5 mL of BHI broth (Oxoid Deutschland GmbH, Wesel, Germany), and the suspension was cultured at 37 °C aerobically. After 24 h, the suspension had a concentration of 10^9^ colony-forming units (CFU)/mL, measured using a dilution series. Keeping the concentration, cells were transferred into MH (Müller–Hinton broth, Becton Dickinson GmbH, Heidelberg, Germany). In preliminary experiments, with different bacterial cell concentrations as inoculum, different media (MH and BHI) at two different concentrations (normal and half-concentrated) were tested in triplicate in our root model. As another important parameter, three different incubation periods of 1–2, 3–4, and 5 weeks were also tested. The rationale underlying this overflow model is (i) that bacteria should persistently have sufficient medium to grow and (ii) that metabolic end products can diffuse to ensure microbial in-canal and in-tubule growth and biofilm formation. Without showing the results for every combination (broth/broth strength/incubation period), the following protocol was found to be optimal and was consequently used for the main experiment. The overflow model with a load of 200 µL of 0.5 × (half-strength to reduce growth speed) MH medium, inoculation with 15 µL of an *E. faecalis* suspension of 10^9^ CFU/mL MH, and incubation for 3–4 weeks aerobically at 37 °C (Figure 8) was considered ideal. Importantly, the medium was refreshed three times a week for an optimized growth dynamic. For refreshing, all consumed MH medium was removed using a syringe, and approximately 200 µL of fresh 0.5 × MH medium was reloaded. Settled or invasive bacteria were of course not removed by this procedure.

### 4.4. Centrifugation Model (Process B)

**Figure 9:**
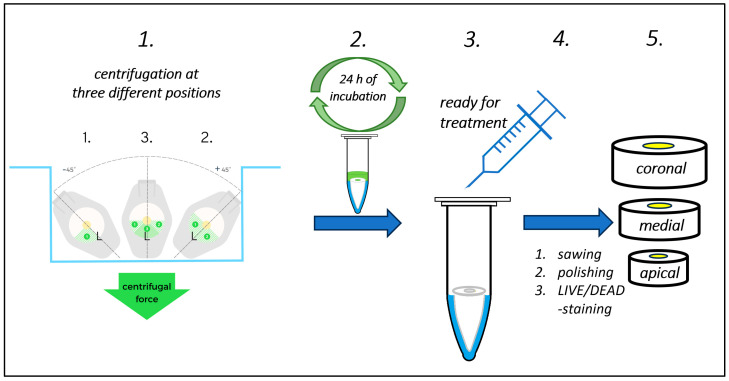
Schematic representation of the centrifugation model for inoculation of roots and subsequent procedures for treatment evaluation and visualization (Process B with steps 1–5). Step 1: The roots were centrifuged at 275× *g* and then at 2250× *g* for 2 min each. In order to achieve the greatest possible number of inoculated (“infected”) tubules, the centrifugation process was carried out in three different positions (−45°, 0°, +45°). The final centrifugation process was in the lingual/oral (L) direction. The bacterial suspension was renewed between each centrifugation procedure. Step 2: Medium was added, and the tubes were incubated at 37 °C for 24 h aerobically to stimulate intratubular bacterial growth as well as deeper penetration of *E. faecalis*. Steps 3–5, same as Figure 8.

The protocol used for bacterial inoculation by centrifugation in the present study is a modification of the protocol described by Ma et al. (2011) [15]. At a load of 100 µL (10^8^ CFU) of the *E. faecalis* stock (10^9^ CFU/mL MH), the roots placed in Eppendorf tubes were centrifuged at 275× *g* (or rcf) and then at 2250× *g* for 2 min each. In order to achieve the greatest possible number of inoculated (“infected”) tubules, the centrifugation process was carried out in three different positions (−45°, 0°, +45°), as shown in Figure 9. Between each centrifugation procedure, the bacterial suspension was renewed (liquid removed and 100 µL suspension loaded). Any residual MH supernatant was removed. Finally, 100 µL of fresh, sterile MH was loaded, and the tubes were incubated at 37 °C for 24 h aerobically to stimulate intratubular bacterial growth as well as deeper penetration of *E. faecalis*. Particularly important here was the localization of accessible tubules and the correct positioning of the teeth in the centrifuge. Uniform, deep, and satisfactory inoculation with bacteria by centrifugation is hindered by any sclerosis within the tubules. That is why only nonsclerotized extracted wisdom teeth were eligible for Process B (SCG0), with the exclusion of sclerotized teeth (SCG1-3).

### 4.5. Grouping and Treatment

After pretreatment using protocols Pr1 or Pr2 and inoculation by overflow (process A) or centrifugation (process B), the *E. faecalis*-loaded specimens were divided into nine groups, listed in Table 3, to demonstrate the effect of different disinfection treatments. The nine groups reflected three different treatments tested on three different combinations of pretreatment/inoculation. The treatment protocols can be described as follows:

1. Control: No treatment.
2. Conventional rinsing: Samples were irrigated with the help of a 27-G side-vented needle (Covidien, Dublin, Ireland) with 2 mL EDTA (17%) for 60 s, followed by 5 mL NaOCl (3%) for 60 s and 5 mL physiological NaCl for 60 s in order to dilute NaOCl traces.
3. Rinsing with sonic activation (EDDY): Samples were irrigated with the help of the same needle with 2 mL EDTA (17%) for 60 s as under (2), but followed by 1 mL NaOCl (3%) for 15 s, sonic activation (5000–6000 Hz) with EDDY for 30 s, irrigation again with 1 mL NaOCl (3%) for 15 s, repetition of sonic activation, irrigation again with 3 mL NaOCl (3%) for 30 s, and finally 5 mL of physiological NaCl for 60 s in order to dilute NaOCl traces. To avoid contamination, the rinsing needles and EDDY tips were changed between the samples.

### 4.6. Preparation of Samples for Evaluation

After bacterial inoculation and various subsequent endodontic treatments, all samples were further processed in the same way (see Figure 8, steps 4–5): the roots were cut perpendicularly to the longitudinal axis under water cooling with a rotating diamond saw (cut-grinder Primus, Walter Messner GmbH, Oststeinbek, Germany). This resulted in three slices—one each for coronal, medial, and apical—with a thickness of 3 mm. In the case of groups G1–G6 (overflow model, process A), the resin layer was removed from the surface of the slabs. As examples, a few longitudinal slices were obtained for an overview from a few extra teeth. Preliminary tests showed that surface polishing resulted in more brilliant images. The samples were therefore polished from the upper side with silicium carbide grinding discs with grain sizes of 1200, 2400, and 4000 µm (Waterproof Silicon Carbide Paper FEPA, Struers, Champigny-sur-Marne, France used on EXAKT 400CS, EXAKT Advanced Technologies GmbH, Norderstedt, Germany) before LIVE/DEAD staining. The samples were stained using the FilmTracer^TM^ LIVE/DEAD Biofilm Viability Kit L10316 (Molecular Probes, Eugene, OR, USA) in accordance with the manufacturer’s instructions. The LIVE/DEAD fluorescence solution works with the integrity of the bacterial cell membrane as a parameter. The SYTO 9 green fluorescent nucleic acid stain labels all bacteria in a population independently of viability and membrane integrity. In contrast, the red fluorescent dye propidium iodide (PI) only penetrates dead bacteria or those with damaged membranes. PI shows a stronger affinity for nucleic acids than SYTO 9 and displaces it [38]. SYTO 9 and PI were applied in a 1:1.5 ratio mixture. The specimens have to be kept in the dark during staining and also during transport to the CLSM unit.

### 4.7. Assessment of Sclerosis Grading (SCG)

To improve comparison, samples of different sclerosis grades were almost equally distributed among the experimental groups, assisted by the grading scale presented in Figure 2. Table 3 shows the distribution pattern of sclerosis grade, with n = number of specimens of each grade. However, as some specimens had to be excluded at a later stage, the distribution remained imperfect. Inoculation with bacteria by centrifugation is hindered by any sclerosis. Only SCG0 specimens were therefore eligible for B-Pr2.

### 4.8. Confocal Laser Microscopy Evaluation and Assessment of Treatment

After staining, the samples were carefully washed in ultrapure water for 10 s and then placed in µ-Slide 8-well chambers (ibidi GmbH, Gräfelfing, Germany). Images were acquired using an inverted Zeiss LSM710 confocal laser scanning microscope (Carl Zeiss AG, Oberkochen, Germany) and Zen black acquisition software (version 2.3 SP1). SYTO 9 and propidium iodide were both excited with a 488 nm laser line. The emission was collected using collection windows of either 493–584 nm for SYTO 9 or 604–718 nm for propidium iodide. Overview images of the dental slices in 3 × 3, 4 × 4, or 5 × 5 tile scans, depending on the size of the sample, were taken with a 10× EC Plan-Neofluar objective lens. A 40× LD C-apochromatic water immersion objective lens was used for detailed close-up images. The course of the tubules from the canal entrance to the mantle dentine was exemplified using 9 × 2 tile scans. The samples were divided into 16 equal pieces (segments of a circle, arcs) and analyzed using Zen lite blue (version 3.6. 095.02) software. The sections were measured and evaluated as follows, with the root canal wall being taken as the starting point.

1. The colonization/penetration depth of the living bacteria (green fluorescence) was measured to ensure the presence of *E. faecalis* and to quantify how deeply the bacteria were invading the dentinal tubules.
2. The area of dead bacteria (red fluorescence) and/or diffusion depth of released DNA/RNA (also red) was measured to evaluate the penetration depth of the rinsing solution.

### 4.9. Statistical Analyses

The invasion depth of *E. faecalis* (measured from the root canal wall up to the last viable bacterium detected), the area of bacteria-invaded tubules before treatment, the penetration depth of treatment rinses (measured from the root canal wall up to the last red staining of DNA/RNA), and the area of tubules with released DNA/RNA after treatment were expressed as average values and analyzed using one-way analysis of variance (ANOVA), followed by the least significant difference test using GraphPad Prism version 10.0 (GraphPad Software, Boston, MA, USA).

## 5. Conclusions

In summary, CLSM-guided imaging provides many ways of quantifying endodontic disinfection if the right model is used. It has been shown that the pretreatment protocol A-Pr2 (EDTA only, EDDY-activated) is preferable, as it tends to have the deepest bacterial penetration depth and since the alternative, NaOCl pretreatment (A-Pr1), negatively affects bacterial penetration and also weakens the peritubular dentine structure. In addition, the overflow model is preferable to the centrifugation model in terms of bacterial penetration depth and omnidirectional colonization. It has also been noted that individual anatomical conditions are a major factor influencing colonization and irrigation depths. Anatomic conditions cannot be influenced but can be controlled through specific tooth selection for in vitro studies. The first step in any in vitro study is the appropriate and careful selection of comparable test specimens. This study shows that sclerosis is a hitherto overlooked, independent, and strong influencing factor for the penetration depth of bacteria and—subsequently—irrigation depth. A clear classification of sclerosis grading is therefore needed in order to obtain comparable results. It is difficult to draw conclusions regarding sclerosis grading, and it requires description and selection of test samples. A clinical implication of our results thus could be that the irrigation protocol may need to be adapted to the patient’s age. Additional studies are needed to validate our model’s effectiveness across diverse sample sets and conditions. Also, with regard to the SCG system we introduced in the present study, further studies are needed to validate this classification and verify its effectiveness in the selection of samples for in vitro studies. Our group is working on the translation of our optimized models presented here into clinical applications.

## Figures and Tables

**Table 1:** Heat map of mean values for bacterial colonization/penetration depths (in µm) depending on position (apical to coronal), sclerosis grade (SCG), and three experimental settings (Processes A/B combined with pretreatments Pr1/2).

Control			Position			SCG			
			Coronal	Medial	Apical	0	1	2	3
All		440							
Position	Coronal	680							
	Medial	380							
	Apical	270							
SCG	0	400	520	410	270				
	1	680	900	370	210				
	2	440	520	430	410				
	3	110	170	120	80				
Experiment	B-Pr2	400	520	410	270	400			
	A-Pr2	480	770	380	280		790	420	100
	A-Pr1	420	650	360	250		580	460	110

Bacterial colonization/penetration depths are highlighted using the traffic light method from high (green) to low (red).

**Table 2:** Steps in pretreatment protocols Pr1 and Pr2.

Pr1 (with NaOCl)				Pr2 (without NaOCl)			
	Irrigation/Activation	mL	s		Irrigation/Activation	mL	s
0.	NaOCl between every file	1	15	0.	NaCl between every file	1	15
1.	NaOCl (5%) 40 °C	5	60				
2.	Ultrapure water	5	60	1.	Ultrapure water	5	60
3.	EDTA (17%)	5	60	2.	EDTA (17%)	5	60
4.	Activation with EDDY		30	3.	Activation with EDDY		30
5.	Ultrapure water	5	60	4.	Ultrapure water	5	60
6.	NaOCl (5%), RT	5	60				
7.	Activation with EDDY		30				
8.	Resting phase *		30				
9.	Activation with EDDY		30				
10.	Ultrapure water	5	60				

All irrigants, except initial NaOCl (40 °C, heated on a thermoplate), were used at room temperature (RT) (≈20 °C). For both protocols, specimens were finally autoclaved before inoculation. * exposure to NaOCl.

**Table 3:** Specification of treatment groups including number of specimens, distribution pattern of sclerosis grades (SCG), pretreatment, inoculation model, and disinfection treatments.

Group	N *	SCG (n) **	Pretreatment	Inoculation Process and Model	Treatment
G1	30	SCG1 (10), SCG2 (13), SCG3 (7)	Pr1	A overflow	Control
G2	27	SCG1 (7), SCG2 (13), SCG3 (7)	Pr1	A overflow	Conventional rinsing
G3	30	SCG1 (12), SCG2 (7), SCG3 (11)	Pr1	A overflow	Sonic activation of rinsing (EDDY)
G4	30	SCG1 (9), SCG2 (16), SCG3 (5)	Pr2	A overflow	Control
G5	30	SCG1 (13), SCG2 (10), SCG3 (7)	Pr2	A overflow	Conventional rinsing
G6	30	SCG1 (12), SCG2 (10), SCG3 (8)	Pr2	A overflow	Sonic activation of rinsing (EDDY)
G7	12	SCG0 (12)	Pr2	B centrifugation	Control
G8	12	SCG0 (12)	Pr2	B centrifugation	Conventional rinsing
G9	12	SCG0 (12)	Pr2	B centrifugation	Sonic activation of rinsing (EDDY)

* Number of specimens. ** The demand for SCG0 specimens for Process B (centrifugation) reduced the number of available specimens down to 12 in total. EDDY: sonically powered irrigation (VDW GmbH, Munich, Germany).

## Data Availability

The data presented in this study are available on request from the first (R.M.) and from the corresponding author (G.C.).
